# Phyllotaxis bionics for vortex nanosieves

**DOI:** 10.1038/s41377-021-00641-3

**Published:** 2021-09-22

**Authors:** Min Gu, Yilin Hua, Xinyuan Fang

**Affiliations:** 1 Institute of Photonic Chips, University of Shanghai for Science and Technology, 200093 Shanghai, China; 2 Centre for Artificial-Intelligence Nanophotonics, School of Optical-Electrical and Computer Engineering, University of Shanghai for Science and Technology, 200093 Shanghai, China

**Keywords:** Optical data storage, Nanophotonics and plasmonics

*eLight, 1(5), 2021*

10.1186/s43593-021-00005-9

Simultaneous generation of multiple optical vortex (OV) lies at the heart of the application for orbital angular momentum (OAM) multiplexing both in classical and quantum domains. Previous structure with segmented or interleaved functional sub-areas in a single nano-device has been developed at the cost of its compactness and channel capacity. Back-to-nature design inspired by the spiral phyllotaxis pattern in this work offers a fresh way to fabricate a truly space-degenerated nanoscale multiple-OVs-generator, namely vortex nanosieves. Both free-space and plasmonic near-field OAM multiplexed OVs are produced with two different forms of the phyllotaxis-inspired patterns, which are proved by rigorous mathematical language and completely consistent experimental results. Except for the structural degeneracy of the device, the OAM modes extracted from the vortex nanosieves are controllable according to the internally embedded multiple spirals, which is particularly suitable for the high-order OAM modes generation. Furthermore, the OAM multimode output displays a concentric-ring type naturally with negligible misalignment. These superiorities make the phyllotaxis-inspired vortex-nanosieves device a programmable multi-OAM-modes generator and multiplexer, which will facilitate research in both on-chip photonic devices and OAM information optics.

